# Pre-operative denosumab is associated with higher risk of local recurrence in giant cell tumor of bone: a systematic review and meta-analysis

**DOI:** 10.1186/s12891-020-03294-2

**Published:** 2020-04-20

**Authors:** Xi Chen, Hairui Li, Shibai Zhu, Yiou Wang, Wenwei Qian

**Affiliations:** 1 Department of Orthopedic Surgery, Peking Union Medical College Hospital, Peking Union Medical College, Chinese Academy of Medical Science, Beijing, China; 2 Department of Plastic Surgery, Peking Union Medical College Hospital, Peking Union Medical College, Chinese Academy of Medical Science, Beijing, China

**Keywords:** Giant cell tumor, Denosumab, Recurrence

## Abstract

**Background:**

In 2013, denosumab was introduced as peri-operative adjuvant treatment for giant cell tumor (GCT) of bone as it inhibits osteoclast activity. It is suggested that denosumab relives pain, facilitate curettage in lesions requiring resection initially. However, controversy remains whether denosumab increases the risk of local recurrence after surgery.

**Methods:**

Medline, Embase and the Cochrane Library were comprehensively searched in June 2019 to identify studies investigating the clinical outcome of GCT of bone with and without peri-operative denosumab after surgery. Data were gathered and a meta-analysis was conducted.

**Result:**

Ten studies with 1082 cases (169 in denosumab group, 913 in control group) were included. Overall, denosumab was associated with significantly higher risk of recurrence(*P* < 0.02) and inferior 5 year recurrence free survival(*P* = 0.000). Denosumab and curettage has a relatively higher risk of recurrence comparing to curettage alone(*P* = 0.07). The risk of recurrence is not significantly increased if denosumab was administered both preoperatively and postoperatively(*P* = 0.24).

**Conclusion:**

Administration of denosumab is associated with increased risk of recurrence due to a variety of reasons, though it is proven effective in relieving pain, enabling curettage and improved functional outcome. Post-operative denosumab is recommended as it continuously suppress/eliminate residue tumor cells.

## Background

Giant cell tumor is a benign but locally aggressive bone neoplasm which typically affects young patients in the meta-epiphyseal regions of the bone [1]. The treatment of GCT remains controversial over decades as to search for the balance between adequate surgical margin and sufficient adjacent joint function. Curettage can minimize bone loss and the destruction to soft tissue, thus leading to better joint function, less pain and less peri-operative complications. But it is associated with higher risk of local recurrence [2–4]. Some researches introduced adjuvants including local adjuvant and bisphosphonates combined with curettage, but the results were inconclusive [5–7].

In 2013, the Food and Drug Administration of United States approved the clinical use of denosumab, a human monoclonal antibody that interferes in the bone remodeling process by binding receptor activation of nuclear factor-β ligand (RANKL) to treat osteoporosis, bone metastasis and bone tumors including giant cell tumor [8].

In the past 6 years, studies have been done to investigate the clinical effect of denosumab on giant cell tumor of bone, suggesting that it might be a promising adjuvant therapy by reducing osteoclast activity.

Some studies suggest that denosumab is associated with tumor response, facilitating curettage in lesions that required resection initially by allowing the forming of peripheral bone rim and eventually reduce surgical morbidity [9–11]. However, other studies reported minimal inhibitory effect on GCT from denosumab [12], some argue that denosumab might make it difficult to achieve a clear margin during surgery and therefore increase the risk of recurrence [13].

The incidence of giant cell tumor of bone is quite low, reported incidence included 1.38 per million in US, 1.49 per million in China [14]. Considering the rare incidence and the fact that denosumab has only been approved by FDA for 6 years, most studies investigating on this subject are retrospective studies with small cohort. The purpose of our meta-analysis is to gather existing data from these studies and evaluate the safety and clinical efficacy of denosumab as peri-operative treatment of GCT of bone. The primary aim is to analyze whether denosumab increases the risk of local recurrence after surgery and explore any potential causes.

## Methods

### Search strategy

Medline, Embase and the Cochrane Library were comprehensively searched by two independent researchers until June 31st, 2019. Search terms included: denosumab, giant cell tumor, GCT, surgery and related MeSH terms. The search term used in Medline were (((“Giant Cell Tumor of Bone”[Mesh] OR “Giant Cell Tumors”[Mesh]) OR (“Gene Cell Tissue”[Journal] OR “gct”[All Fields])) OR (“giant cell tumour”[All Fields] OR “giant cell tumors”[MeSH Terms] OR (“giant”[All Fields] AND “cell”[All Fields] AND “tumors”[All Fields]) OR “giant cell tumors”[All Fields] OR (“giant”[All Fields] AND “cell”[All Fields] AND “tumor”[All Fields]) OR “giant cell tumor”[All Fields])) AND (“denosumab”[MeSH Terms] OR “denosumab”[All Fields]).Any additional studies were identified from references of retrieved studies.

### Inclusion and exclusion criteria

Studies were included if they (1) were clinical trials, (2) included patients receiving denosumab and underwent surgery for giant cell tumor of bone, (3) compared clinical outcome with patients that underwent surgery without denosumab treatment. Studies were excluded if they were (1) conference abstracts, non-human studies, cadaveric studies or studies published in forms other than clinical articles, (2) studies without quantitative data, (3) studies without comparison between cases receiving denosumab and cases underwent surgeries alone, or (4) studies that included GCT of the skull.

### Data extraction and quality assessment

Data were collected from included studies and were screened and analyzed by two researchers. Quantitative data and key information in each studies were recorded in Excel and verified. The quality of included studies were assessed according to the Newcastle-Ottawa Scale (NOS). The level of evidence of included studies were assessed based on the CEBM Level of Evidence.

### Statistical analysis

Discontinuous data were analyzed by odds ratios. Pooled analysis was performed with an 95% confidence intervals (CIs). Generally, Mantel-Haenszel(M-H) method was utilized, Peto’s method was applied when event incidence is deemed rare and heterogeneity is low. Heterogeneity was assessed using the chi-squared and I-squared(I^2^)tests. A fixed effect model was applied when I^2^ < 50%, and a random effect model when I^2^ > 50%. A *p* value< 0.05 was considered statistically significant. In the cases that trials have no event in one arm or another, a small quantity (0.5) would be added to the cell counts to avoid division by zero errors according to the Systematic Reviews in Health Care: Meta-Analysis in Context. In the case where the *P* = 0.000 is displayed, it means *P*<0.0005. Revman 5.3 and Stata 14.0 were used to conduct this meta-analysis as Revman provides more detailed forest plot in discontinuous data analysis while Stata is capable of survival analysis and various heterogeneity analysis. Data and results were cross-checked between these two software.

## Results

### Study characteristics

**Fig. 1:**
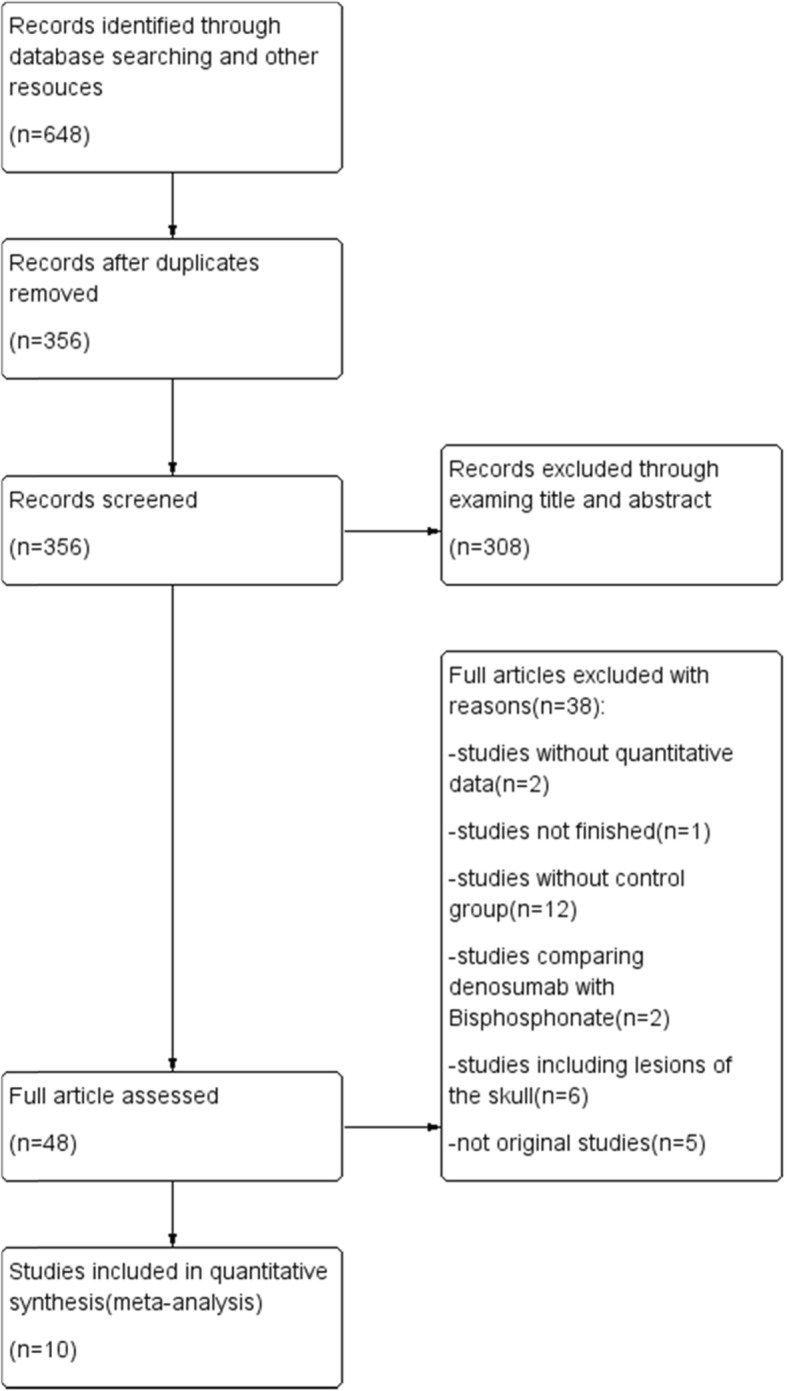
Flow diagram

A comprehensive search of Medline, Embase and the Cochrane Library yielded 648 articles. Ten studies with 1082 cases (169 in denosumab group, 913 in control group) were eventually included after careful screening title, abstract, and full texts by two independent researchers. (Fig. 1). Basic characteristics and quality assessment of included studies are listed in Table 1. Parameters including recurrence, indication to administer denosumab, administration time and dosage, surgical approach, lesion site, primary/recurrent lesion Campannaci grade, denosumab related complication and malignant transformation of lesion are recorded and analyzed.

**Table 1:** Study Characteristics

Study	Year	Country	No. of cases		Follow-up (months)	Site	Surgery	Quality Score
			Denosumab	Control				
Agarwal(19)	2018	India	25	34	12 to 24	E/K	C/W	********
Chen(15)	2018	China	20	10	3 to 36	K	Unspecified	*****
Costantino(3)	2018	Italy	25	222	> 24	E/K	C	******
Liu(18)	2016	China	1	3	> 8	E	C	****
Medellin(20)	2018	UK	7	100	> 12	E	C/W	******
Scoccianti(9)	2018	Italy	12	9	14 to 92	E/K	C	******
Tsukamoto(13)	2019	Japan	25	317	54 to 124	E/K	C/W	******
Urakawa(17)	2018	Japan	40	158	60 to 72	E/K	C	*****
Yang(22)	2018	China	6	10	> 24	K	C/W	********
Zou(21)	2018	China	8	50	21 to 321	K	C/W	******

*E* Extremity; *K* Trunk; *C* Intralesional Curettage; W Wide Resection

Histopathological diagnosis of giant cell tumor was all achieved in included studies. The indications for denosumab are specified in 6 studies, including: lesions in distal radius, invasive radiographic findings, unresectable lesions and possible severe morbidity after surgery.

### Recurrence

**Fig. 2:**
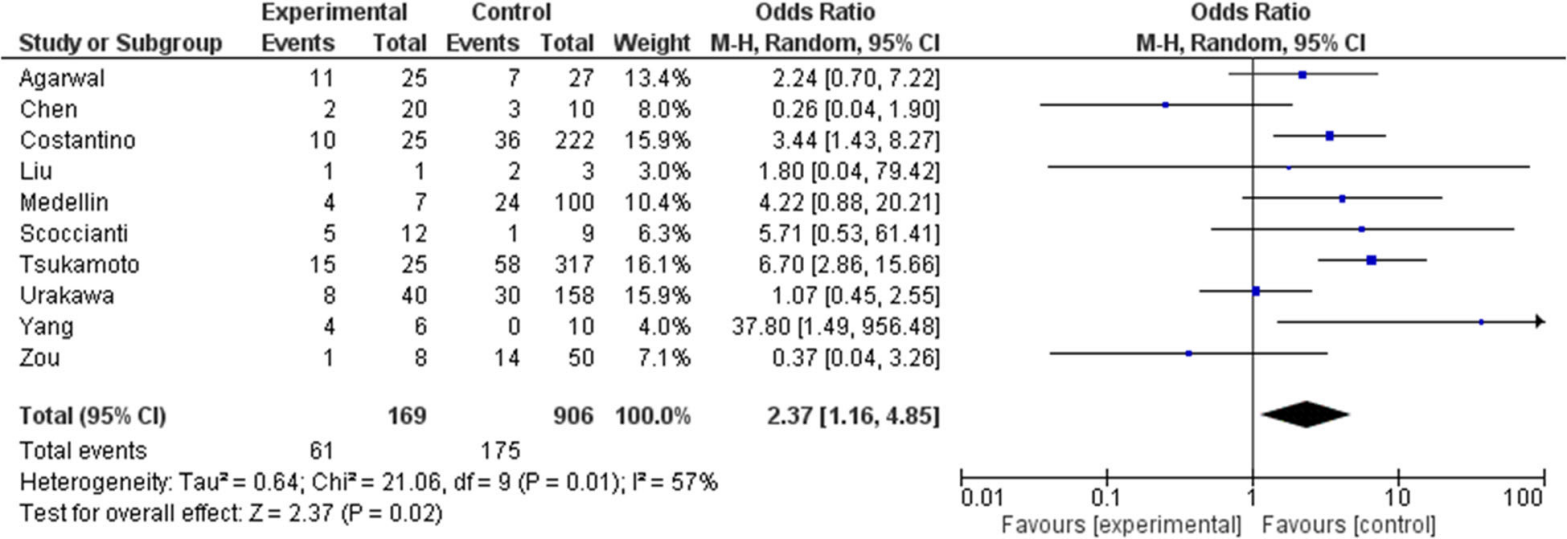
Overall Recurrence

**Fig. 3:**
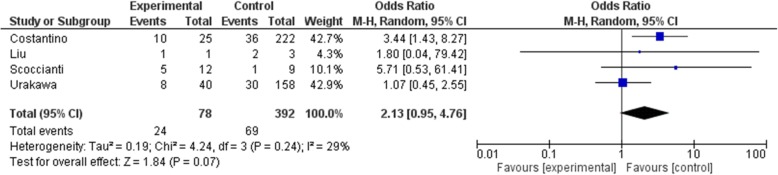
Recurrence in denosumab and curettage versus curettage alone

**Fig. 4:**
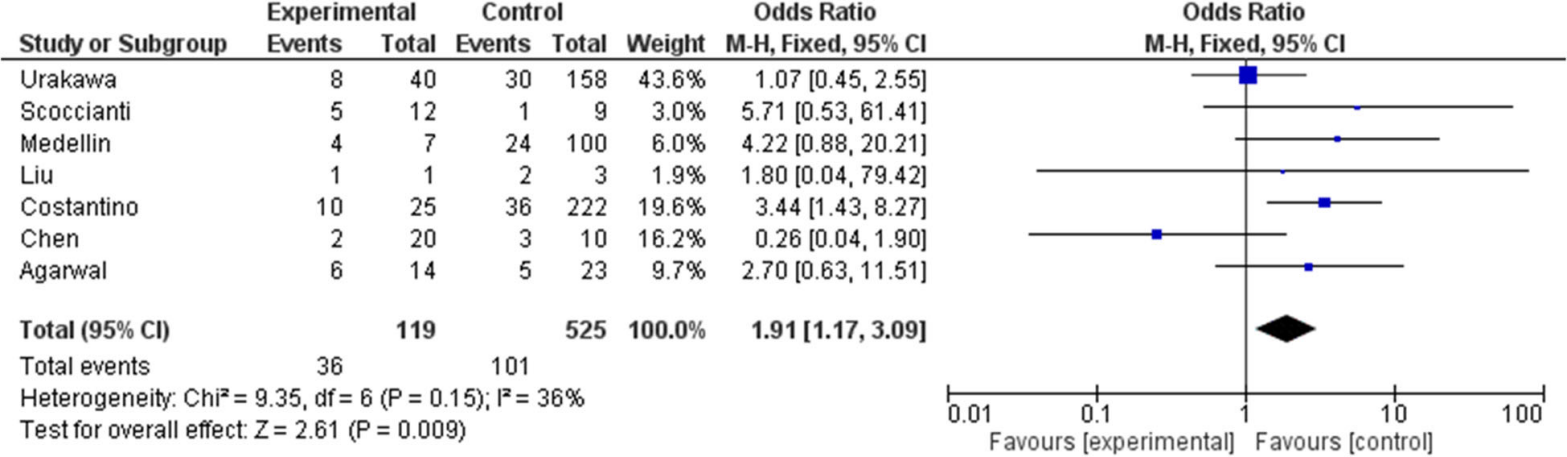
Recurrence with primary lesions

**Fig. 5:**
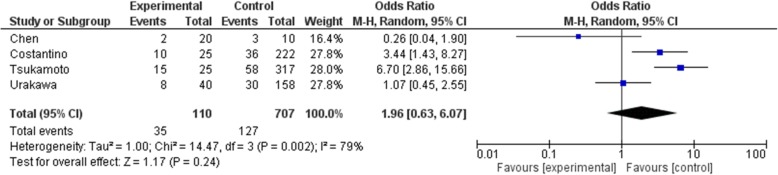
Preoperative and postoperative denosumab versus control

As the primary outcome of this meta-analysis, local recurrence is recorded in all included studies. Administration dosage was 120 mg per injection and interval was once a month with 0–3 additional dosage in the first month. The total dosage given prior to surgery was inconsistent among included studies. Four studies [13, 15–17] administered denosumab pre-operatively and post-operatively. Overall, 61 cases in the denosumab group and 175 cases in the control group had local recurrence. Pooled analysis showed significantly higher risk of recurrence in the denosumab group (OR: 2.37, 95% CI: 1.16 to 4.85, *p* = 0.02, I^2^ = 57%)(Fig. 2). Surgical choice was taken into account by comparing cases underwent curettage with denosumab and curettage alone in 4 studies [9, 16–18](OR: 2.13, 95% CI: 0.95 to 4.76, *p* = 0.07, I^2^ = 29%)(Fig. 3), though the result was not statistically significant. Seven studies [9, 15–20] reported outcome on primary lesions, which also suggest significantly higher risk of local recurrence in the denosumab group (OR: 1.91, 95% CI: 1.17 to 3.09, *p* = 0.009, I^2^ = 36%)(Fig. 4). In 4 studies [13, 15–17] denosumab was administered both preoperatively and postoperatively, no statistically significant difference was found when comparing pre and post denosumab administration group and control group in terms of local recurrence (OR: 1.96, 95% CI: 0.63 to 6.07, *p* = 0.24, I^2^ = 79%)(Fig. 5).

### Recurrence free survival

Recurrence free survival analysis was conducted in 4 studies [13, 16, 20, 21], pooled analysis including 754 cases with at least 12 months of follow-up showed that denosumab is associated with unfavorable 5 year recurrence free survival (ES: 3.707, 95% CI: 2.30 to 5.98, *p* = 0.000, I^2^ = 0%).

### Complications and others

Six studies [9, 13, 15, 19, 21, 22] reported denosumab-related complications, in the 388 cases treated with denosumab, no osteo-necrosis of jaw or hypocalcemia was observed. One study [13] reported a periapical abscess and a periodontal disease during the course of denosumab administration. No other denosumab-related complications were reported in all included studies. Prior to administration of denosumab, dental check, blood test for renal function and calcium level were conducted by Agarwal et al. [19]. Scoccianti et al. [9] conducted dental check before denosumab administration. Oral supplementary of Vitamin D and calcium is administered in 4 studies [3, 9, 13, 19]. Generally, Vitamin D was given more than 400 IU/day and calcium more than 500 mg/day. Administration of denosumab were similar, most studies administered denosumab once a month with 1–3 additional doses during the first month, the duration of administration prior to surgery differs for various reasons. Yang et al. [22] reported significant decreased VAS score, intraoperative blood loss and CT enhancement after administration.

### Radiographic outcome

#### Publication bias

**Fig. 6:**
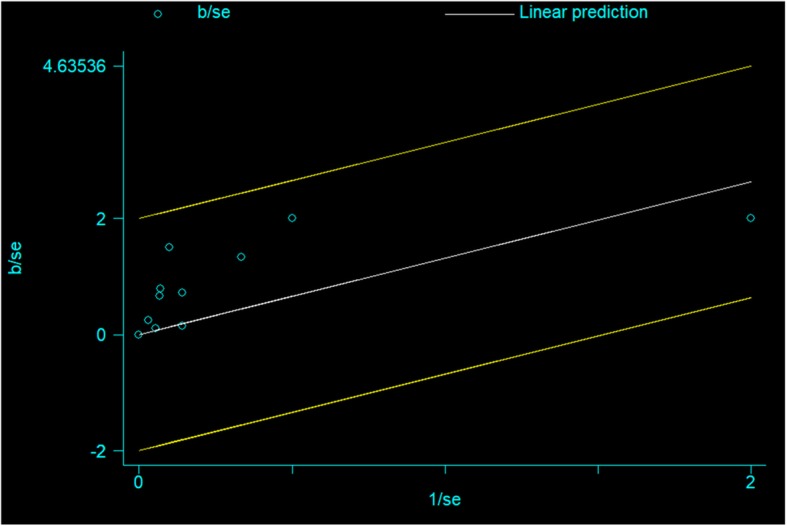
Galbraith plot-overall recurrence

**Fig. 7:**
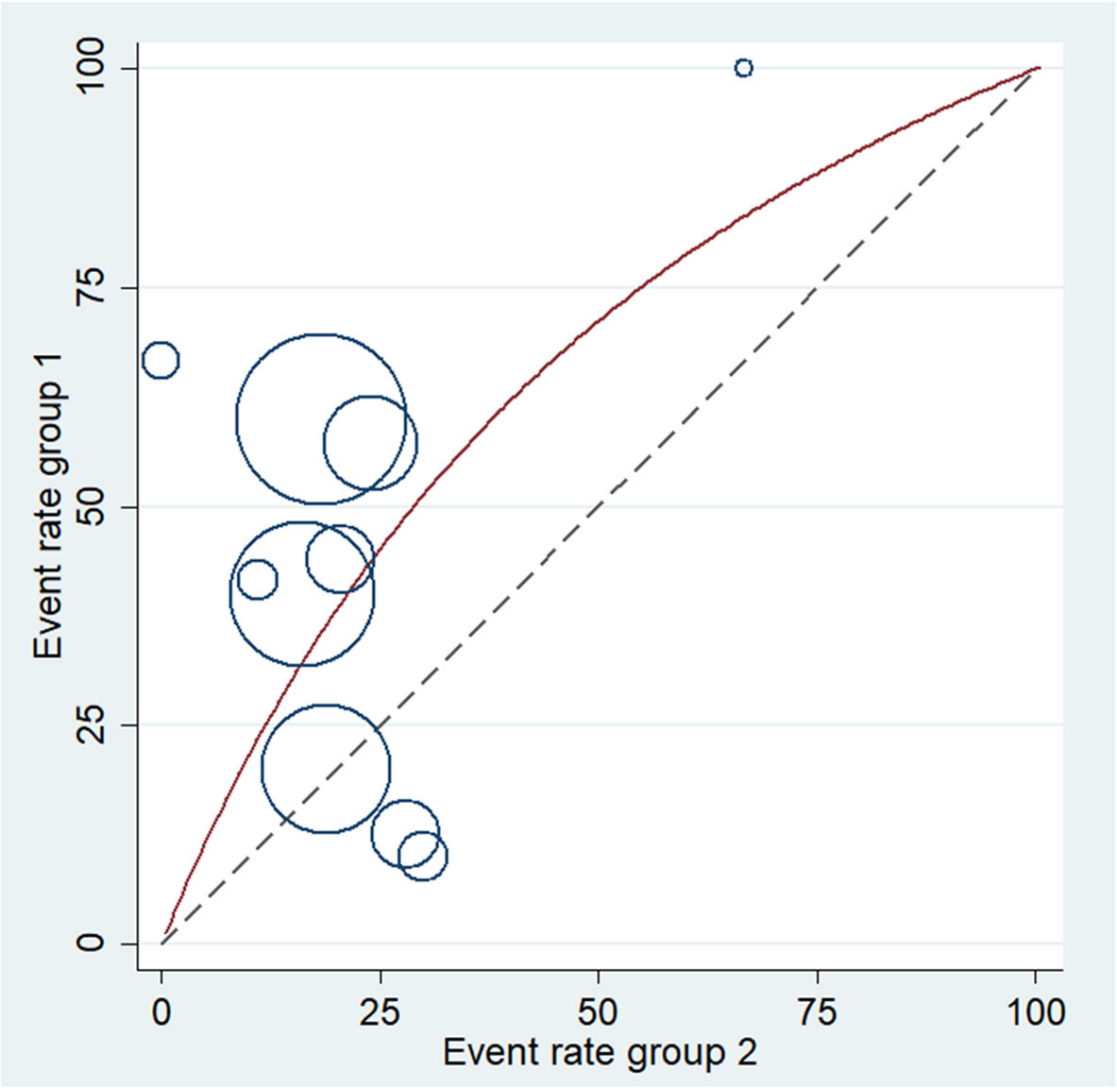
L’abbe plot-overall recurrence

Galbraith test (Fig. 6) and L’abbe test (Fig. 7) was conducted to assess heterogeneity among different studies in terms of local recurrence, which showed low heterogeneity among different studies.

#### Sensitivity analysis

Sensitivity analysis was conducted by excluding study with fewer than 10 cases and studies with less than 12 months of follow up, which did not have a significant impact on our result in terms of local recurrence.

## Discussion

In vitro studies have found that denosumab suppress the proliferation of giant cells by actively inhibits osteoclast activity [12], a reduction in the stroma cells was also observed by Chawla et al. [10]. Osteolysis is then suppressed and new bone formation innovated [23].

However, controversy remains regarding to the oncologic outcome of giant cell tumor of bone after denosumab treatment. A total of 169 cases underwent denosumab treatment and surgery, 61(36.1%) of them experienced recurrence, pooled analysis of overall recurrence and recurrence free survival showed a significantly inferior outcome in the denosumab group. The following factors are considered relevant to the higher recurrence rate in the denosumab group:

It is has been reported that the neoplastic cells turned proliferative once the use of denosumab is stopped [12], which may lead to recurrence if adequate surgical margin is not achieved. Several studies have reported the newly formed sclerotic bone and thickened cortex made it difficult to identify surgical margin and to curette. Residue tumor cells may hide within the new bone and thickened cortex and may recur once denosumab is discontinued [22, 24, 25]. Traub et al. [25] described that the tumor tissue was replaced by gritty, fibrous-like tissue new bone, they suggested intra-operative fluoroscopy to guide the extent of resection. On the other hand, it is argued that the newly formed peripheral bone rim and hardened tumor tissue reduced the risk of inadvertent contamination [19]. Although we did not acquire sufficient data to conduct pooled-analysis on the outcome of denosumab combining curettage and local adjuvant, it is strongly recommended in many studies to use local adjuvant and high speed burr [3, 24, 26, 27]. Most studies included in our analysis applied various methods of local adjuvant in most patients except for one study specified that no local adjuvant was used [20].

**Fig. 8:**
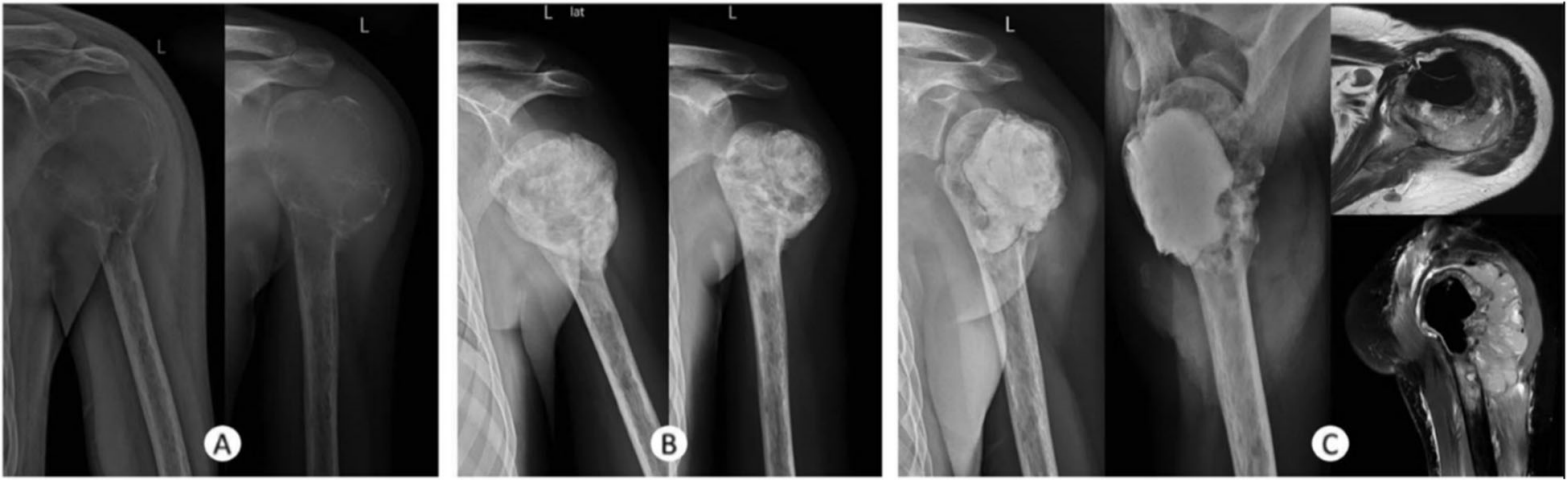
**a**-**c** This shows a patient with a proximal humerus GCT with (**a**) a radiograph at presentation who was treated with eight injections of denosumab, which led to (**b**) bony shell formation and sclerosis within the lesion after which the patient underwent intralesional surgery for disease clearance. Eight months postoperatively the patient presented with a local recurrence, which as seen on (**c**) radiography and MRI suggested aggressive malignant transformation. Biopsy confirmed osteosarcoma

Giant cell tumor of bone typically presents as an eccentric and radiolucent lesion on radiograph, sclerosis can be seen in some lesions. Cortical discontinuity can be found on X-ray and CT, and extensive edema can be found on MRI, which are considered as prognostic markers for local recurrence [6, 28]. Sclerosis around and within the lesion tumor after denosumab was reported in most studies. A significant decrease in enhancement in enhanced CT value suggested the decrease of blood supply after denosumab administration [22]. On MRI, marrow replacement by tissue with different signal intensity were reported [18]. For 18F-FDG PET/CT, studies found 94–96% cases underwent denosumab treatment had decreased SUVmax values [10, 29]. Ueda et al. [11] used CT/MRI and 18FDG–PET/PET–CT to assess objective response of giant cell tumor of bone after denosumab treatment and found a 35%/82%/71% response rate according to RECIST/EORTC/Choi Criteria respectively, suggesting a robust radiographic response after denosumab treatment. Images of GCT of bone on X-ray reported by Agarwal et al. [19] was cited in Fig. 8 upon permission from editorial office.

Surgical choice between intralesional curettage and wide resection might influence the risk of recurrence [16]. Previous studies have reported a significantly lower risk of recurrence after wide resection than curettage [2, 3]. Through pre-operative administration of denosumab intralesional curettage became feasible in lesions requiring wide resection originally [3]. Pooled analysis compared recurrence between denosumab before curettage and curettage alone, which showed a tendency towards increased risk of recurrence in the denosumab group, but the result was not statistically significant considering only limited number of studies and cases were involved. This can be explained in that these lesions were more aggressive originally and would receive more radical surgical procedures if denosumab was not applied.

In most studies denosumab was preferred in lesions that are either large, located in distal radius (lesions located in distal radius are considered more aggressive and more likely to recur [21]), with higher Campanacci grade or have other progressive signs, which inherently pose a greater risk of recurrence. Agarwal et al. [19] matched the denosumab group and control group in terms of lesion size and Camapannaci grade, 11 of 25(44%) cases in the denosumab group had local recurrence comparing to 7 of 34(21%) cases in the control group, though the difference was not statistically significant due to limited sample size. A randomized controlled trial is currently being conducted in Japan [26], which could decrease the influence of selection bias. However,due to the rare incidence of this disease, such investigation can be very time consuming. Pooled analysis combining recurrent and primary tumor yielded similar result comparing to that of primary tumor alone, which is in consistent with previous finding that recurrent tumors were not at increased risk for recurrence [27].

Standard administration of denosumab included monthly injection with 1–3 additional dosage in the first month, which was applied in all included studies. There is no consensus on how many doses should be given prior to surgery as many factors including radiographic evaluation, economy and patients’ choice are taken into account. Agarawal et al. [19] proposed three weekly doses were enough to solidify the tumor and decrease spillage. Chen et al. [15] recommend the duration of medication should be shortened to the greatest extent to avoid formation of thick rim of new bone. Urakawa et al. [17] applied denosumab both pre-operatively and post-operatively, in the 31 cases treated with denosumab, administration exceeding 5 times was significantly associated with lower risk of recurrence. When only studies applying preoperative and postoperative denosumab are included, pooled analysis showed no significant difference in recurrence comparing to the control group. It might be deduced that postoperative denosumab continues suppress/eliminate any residue tumor cells in the body.

This meta-analysis has several limitations: 1.Due to the rare incidence of the disease, all included studies were retrospective studies with small cohort, hence the quality of our study is limited and further analysis based on anatomic site was not feasible. 2.Cases were not randomized or standardize between denosumab group and control group, which created selection bias. Most studies applied denosumab to more aggressive lesions. 3.Follow up in included studies were not in consistent with each other, which could influence our assessment of local recurrence. 4. Although sclerosis and ossification is observed in most studies, we did not acquire sufficient data to conduct pooled analysis to assess radiographic outcome after denosumab treatment.

Our study has several strength:1. Due to the rare incidence (1–1.5 per million) [14] of giant cell tumor and the fact that denosumab has only been introduced to treating giant cell tumor for nearly a decade, there has been limited number of studies and cases reported on this issue. Our analysis gathered existing and valuable information to conduct a quantitative analysis with a relatively large number of cases involved. 2.Articles were screened carefully by two independent researchers. 3.Attrived data were verified and sorted in subgroups to analyze any potential causes of the difference in recurrence. 4.Statistical analysis was conducted with rigorous heterogeneity assessment. 5.Considering the rare incidence of denosumab-induced complication, we gathered a rather large cohort to investigate this issue.

## Conclusion

As a benign tumor, the treatment of giant cell tumor of bone has been focusing more on relieving pain, optimizing functional outcome and minimizing surgical trauma. Denosumab has been shown effective in relieving pain. Pre-operative denosumab enabling curettage of lesions required resection initially. However, as an aggressive lesion, reported recurrence of giant cell tumor of bone following curettage ranged from 14 to 34% [3, 6, 16, 27, 30–32]. In our analysis, we have found a significant higher recurrence when patients are treated with denosumab prior to surgery. We have also found that the risk of recurrence is not significantly increased if denosumab is administered both pre-operatively and post-operatively. Pre-operative and post-operative denosumab combining with curettage when adequate surgical margin is carefully achieved might be an appropriate option. Further studies with longer follow-up and rigorous design is needed to further investigate the clinical efficacy and safety of denosumab on the treatment of giant cell tumor of bone.

## Data Availability

Not applicable. The data used for analysis was retrieved from openly published studies listed in our manuscript.

## Abbreviations

GCT: Giant cell tumor

## References

[1] Campanacci M, Baldini N, Boriani S, Sudanese A (1987). Giant-cell tumor of bone. J Bone Joint Surg Am.

[2] Klenke FM, Wenger DE, Inwards CY, Rose PS, Sim FH (2011). Giant cell tumor of bone: risk factors for recurrence. Clin Orthop Relat Res.

[3] Errani C, Ruggieri P, Asenzio MA, Toscano A, Colangeli S, Rimondi E, Rossi G, Longhi A, Mercuri M (2010). Giant cell tumor of the extremity: a review of 349 cases from a single institution. Cancer Treat Rev.

[4] van der Heijden L, Dijkstra PD, van de Sande MA, Kroep JR, Nout RA, van Rijswijk CS, Bovee JV, Hogendoorn PC, Gelderblom H (2014). The clinical approach toward giant cell tumor of bone. Oncologist.

[5] Prosser GH, Baloch KG, Tillman RM, Carter SR, Grimer RJ (2005). Does curettage without adjuvant therapy provide low recurrence rates in giant-cell tumors of bone?. Clin Orthop Relat Res.

[6] Balke M, Schremper L, Gebert C, Ahrens H, Streitbuerger A, Koehler G, Hardes J, Gosheger G (2008). Giant cell tumor of bone: treatment and outcome of 214 cases. J Cancer Res Clin Oncol.

[7] van der Heijden L, van de Sande MA, van der Geest IC, Schreuder HW, van Royen BJ, Jutte PC, Bramer JA, Oner FC, van Noort-Suijdendorp AP, Kroon HM (2014). Giant cell tumors of the sacrum--a nationwide study on midterm results in 26 patients after intralesional excision. Eur Spine J.

[8] Gaston CL, Grimer RJ, Parry M, Stacchiotti S, Dei Tos AP, Gelderblom H, Ferrari S, Baldi GG, Jones RL, Chawla S (2016). Current status and unanswered questions on the use of Denosumab in giant cell tumor of bone. Clin Sarcoma Res.

[9] Scoccianti G, Totti F, Scorianz M, Baldi G, Roselli G, Beltrami G, Franchi A, Capanna R, Campanacci DA (2018). Preoperative Denosumab with curettage and Cryotherapy in Giant cell tumor of bone: is there an increased risk of local recurrence?. Clin Orthop Relat Res.

[10] Chawla S, Henshaw R, Seeger L, Choy E, Blay JY, Ferrari S, Kroep J, Grimer R, Reichardt P, Rutkowski P (2013). Safety and efficacy of denosumab for adults and skeletally mature adolescents with giant cell tumour of bone: interim analysis of an open-label, parallel-group, phase 2 study. Lancet Oncol.

[11] Ueda T, Morioka H, Nishida Y, Kakunaga S, Tsuchiya H, Matsumoto Y, Asami Y, Inoue T, Yoneda T (2015). Objective tumor response to denosumab in patients with giant cell tumor of bone: a multicenter phase II trial. Ann Oncol.

[12] Mak IW, Evaniew N, Popovic S, Tozer R, Ghert M (2014). A translational study of the neoplastic cells of Giant cell tumor of bone following Neoadjuvant Denosumab. J Bone Joint Surg Am.

[13] Tsukamoto S, Mavrogenis AF, Leone G, Righi A, Akahane M, Tanzi P, Kido A, Honoki K, Tanaka Y, Donati DM (2019). Denosumab does not decrease the risk of lung metastases from bone giant cell tumour. Int Orthop.

[14] Liede A, Hernandez RK, Tang ET, Li C, Bennett B, Wong SS, Jandial D (2018). Epidemiology of benign giant cell tumor of bone in the Chinese population. J Bone Oncol.

[15] Chen Z, Yang Y, Guo W, Yang R, Tang X, Yan T, Ji T, Xie L, Xu J, Wang J (2018). Therapeutic benefits of neoadjuvant and post-operative denosumab on sacral giant cell tumor: a retrospective cohort study of 30 cases. J BUON.

[16] Errani C, Tsukamoto S, Leone G, Righi A, Akahane M, Tanaka Y, Donati DM (2018). Denosumab may increase the risk of local recurrence in patients with Giant-cell tumor of bone treated with curettage. J Bone Joint Surg Am.

[17] Urakawa H, Yonemoto T, Matsumoto S, Takagi T, Asanuma K, Watanuki M, Takemoto A, Naka N, Matsumoto Y, Kawai A (2018). Clinical outcome of primary giant cell tumor of bone after curettage with or without perioperative denosumab in Japan: from a questionnaire for JCOG 1610 study. World J Surg Oncol.

[18] Liu C, Tang Y, Li M, Jiao Q, Zhang H, Yang Q, Yao W (2016). Clinical characteristics and prognoses of six patients with multicentric giant cell tumor of the bone. Oncotarget.

[19] Agarwal MG, Gundavda MK, Gupta R, Reddy R (2018). Does Denosumab change the Giant cell tumor treatment strategy? Lessons learned from early experience. Clin Orthop Relat Res.

[20] Medellin MR, Fujiwara T, Tillman RM, Jeys LM, Gregory J, Stevenson JD, Parry M, Abudu A (2018). Prognostic factors for local recurrence in extremity-located giant cell tumours of bone with pathological fracture. Bone Joint J.

[21] Zou C, Lin T, Wang B, Zhao Z, Li B, Xie X, Huang G, Yin J, Shen J (2019). Managements of giant cell tumor within the distal radius: a retrospective study of 58 cases from a single center. J Bone Oncol.

[22] Yang Y, Li Y, Liu W, Xu H, Niu X (2018). A nonrandomized controlled study of sacral giant cell tumors with preoperative treatment of denosumab. Medicine (Baltimore).

[23] Branstetter DG, Nelson SD, Manivel JC, Blay JY, Chawla S, Thomas DM, Jun S, Jacobs I (2012). Denosumab induces tumor reduction and bone formation in patients with giant-cell tumor of bone. Clin Cancer Res.

[24] Muller DA, Beltrami G, Scoccianti G, Campanacci DA, Franchi A, Capanna R (2016). Risks and benefits of combining denosumab and surgery in giant cell tumor of bone-a case series. World J Surg Oncol.

[25] Traub F, Singh J, Dickson BC, Leung S, Mohankumar R, Blackstein ME, Razak AR, Griffin AM, Ferguson PC, Wunder JS (2016). Efficacy of denosumab in joint preservation for patients with giant cell tumour of the bone. Eur J Cancer.

[26] Urakawa H, Mizusawa J, Tanaka K, Eba J, Hiraga H, Kawai A, Nishida Y, Hosaka M, Iwamoto Y, Fukuda H (2019). A randomized phase III trial of denosumab before curettage for giant cell tumor of bone: Japan clinical oncology group study JCOG1610. Jpn J Clin Oncol.

[27] Becker WT, Dohle J, Bernd L, Braun A, Cserhati M, Enderle A, Hovy L, Matejovsky Z, Szendroi M, Trieb K (2008). Local recurrence of giant cell tumor of bone after intralesional treatment with and without adjuvant therapy. J Bone Joint Surg Am.

[28] Turcotte RE, Wunder JS, Isler MH, Bell RS, Schachar N, Masri BA, Moreau G, Davis AM (2002). Giant cell tumor of long bone: a Canadian sarcoma group study. Clin Orthop Relat Res.

[29] Boye K, Jebsen NL, Zaikova O, Knobel H, Londalen AM, Trovik CS, Monge OR, Hall KS (2017). Denosumab in patients with giant-cell tumor of bone in Norway: results from a nationwide cohort. Acta Oncol.

[30] Gaston CL, Bhumbra R, Watanuki M, Abudu AT, Carter SR, Jeys LM, Tillman RM, Grimer RJ (2011). Does the addition of cement improve the rate of local recurrence after curettage of giant cell tumours in bone?. J Bone Joint Surg Br.

[31] van der Heijden L, van der Geest IC, Schreuder HW, van de Sande MA, Dijkstra PD (2014). Liquid nitrogen or phenolization for giant cell tumor of bone?: a comparative cohort study of various standard treatments at two tertiary referral centers. J Bone Joint Surg Am.

[32] Gao ZH, Yin JQ, Xie XB, Zou CY, Huang G, Wang J, Shen JN (2014). Local control of giant cell tumors of the long bone after aggressive curettage with and without bone cement. BMC Musculoskelet Disord.

